# Seed mass of angiosperm woody plants better explained by life history traits than climate across China

**DOI:** 10.1038/s41598-017-03076-2

**Published:** 2017-06-02

**Authors:** Jingming Zheng, Zhiwen Guo, Xiangping Wang

**Affiliations:** College of Forestry, Beijing Forestry University, Beijing, 100083 China

## Abstract

Seed mass is a basic trait in studies of functional ecology. Examining how seed mass is affected by biotic and abiotic factors could improve our understanding of ecological strategies in plants. Here we examined the relationships of seed mass with 13 climate variables and seven life history traits, and partitioned the relative effects of life history traits vs. climate, based on seed mass data for 1265 woody angiosperm species in China. Our results showed that seed mass decreased with latitude, and most climate variables were positively correlated with seed mass. Geographic seed mass pattern was affected by both energy and water availability in the growing season, but the effect of energy availability was more important. Seed mass was also significantly related to other traits such as growth form, fruit type, dispersal mode, breeding system, leaf habit, fruit development time, and minimum juvenile period, with growth form and dispersal mode being the most closely related traits. Our results showed that climate explained much less variation in seed mass than life history traits, and that phylogeny played an important role in shaping the large-scale patterns of seed mass.

## Introduction

Seed size is one of the key traits related to many aspects of plant ecology, and may influence species distribution, population dynamics, and community structure^1^. It’s widely observed that seed mass displays great variations among species and regions, and extant seed plants worldwide have seed masses varying over 11 orders of magnitude^2^. At large spatial scales, it has long been suggest that seed mass decreases with latitude^3, 4^. This latitudinal pattern has not only been found for cross-species studies across regions^5, 6^ and for single-species studies over natural ranges^7, 8^, but also confirmed by recent global studies^5, 9^. Molles *et al*. (2007), using data consisted of 7629 plant species around the world, showed that seed mass exhibited a striking global pattern, with a 320-fold decline in geometric mean seed mass between the equator and 60°^9^. Many studies have examined the drivers of latitudinal seed mass patterns; climate, net primary productivity (NPP), vegetation type, growth form, dispersal mode, and evolution history have been suggested as important factors^8–13^. While these studies have greatly improved our understandings of large-scale gradients in seed mass, the rich flora of China has so far been poorly represented in previous global and regional analyses, which may prevent a full vision of the mechanisms underlying global seed mass pattern.

China is one of the mega-diversity countries in the world with rich endemic species^14, 15^. It covers a latitudinal range from 3 °N to 53 °N and a longitudinal range from 73 °E to 135 °E, and possesses the largest altitudinal gradient in the world^16, 17^. With a great climatic gradient, China is the only country in the world that contains various biomes, from alpine tundra to tropical forests, and from humid forests to supper-arid deserts^17^. Consequently, China provides a unique opportunity to test the hypotheses on large-scale patterns of seed mass. For instance, it is of interest to know if there is also a latitudinal decrease in seed mass across China, as observed in global studies^5,9^? Previous studies have suggest that seed mass increases with temperature, precipitation and NPP^9, 12^. Would this pattern also occur in China? Moreover, as most previous studies did not consider seasonal climate indices in the analysis, it thus remains unclear whether the latitudinal seed mass patterns are more affected by temperature and precipitation in the summer, or more related to climate in the winter. The global analysis by Moles *et al*. (2007) included NPP but not climate as predictors, thus the relative importance of climate vs. NPP in explaining seed mass also remains unclear. Meanwhile, many studies (especially earlier ones) did not examined the role of phylogeny on latitudinal seed mass patterns. However, recent studies have increasingly suggested the necessity to include phylogeny in studies including many taxa^5, 9, 10^. The hyper-diversity of China’s flora has been widely suggested to be related to the unique evolutionary history of the region. Therefore, we postulate that phylogeny plays an important role in affecting the latitudinal seed mass pattern in China. We tested this hypothesis using the phylogenetic generalized least square method (PGLS).

In addition to the abiotic gradients such as climate, variations in seed mass may also be related to the co-variation in life history traits^2, 12, 18^. Previous studies found that growth form, height and dispersal mode were closely associated with seed mass with R^2^s of 0.19~0.40^9, 19, 20^. Other traits have been less studied, but specific leaf area^19^, length of fruit development period^5^, minimum juvenile period^20^, and breeding system^21, 22^ have been shown to be related to seed mass in local scale studies. Some of the reproductive traits, such as fruit type and minimum juvenile period, are important for the life history of woody plants with long lifespan and thus may also influence seed mass. However, many reproductive traits have seldom been included in large scale studies to explain seed mass pattern. In the global analysis by Moles *et al*. (2007), growth form, vegetation type, dispersal characteristics and NPP together accounted for 51% of the variations in seed mass. It is possible that the unexplained variations were caused by other life history traits. Here we compiled a large dataset on seed mass across China, which included not only climate variables but also various life history traits. This provides us a unique opportunity to examine whether seed mass can be better explained by additional traits not considered in previous large-scale studies, and to examine the relative influence of climate vs. life history traits on latitudinal pattern of seed mass.

In this study, we compiled data of seed mass for 1265 woody species, about 15% of the total woody species in China. We also collected information on seven life history traits for these species, including growth form, leaf habit, dispersal mode, fruit type, fruit development days, minimum juvenile period, and breeding system (Table 1). Specifically, we addressed the following questions: 1) How does seed mass change with latitudinal and climate gradients in China, and is the pattern in China similar to those reported in previous global analyses? 2) Amongst the factors of energy availability (e.g. temperature in the growing season), winter temperature, water availability, and gradient of NPP, what are the major climatic correlates of seed mass? 3) Is there a close association between seed mass and life history traits, especially the reproductive traits (e.g. fruit type, fruit development days, minimum juvenile period and breeding system) that are rarely being considered in previous large-scale studies? 4) What are the relative importance of climate vs. life history traits in explaining the seed mass pattern across China?

**Table 1:** Traits and environmental factors examined in this study.

	Unit	No. of values	Description
**Geography gradient**			
Maximum longitude(MALO)	degree	1055	Maximum longitude of a species’ distribution area
Minimum longitude(MILO)	degree	1055	Minimum longitude of a species’ distribution area
Midpoint longitude(MPLO)	degree	1055	Midpoint longitude of a species’ distribution area
Maximum latitude(MALA)	degree	1055	Maximum latitude of a species’ distribution area
Minimum latitude(MILA)	degree	1055	Minimum latitude of a species’ distribution area
Midpoint latitude(MPLA)	degree	1055	Midpoint latitude of a species’ distribution area
**Thermal index**			
MAT	°C	1055	Mean annual temperature
ABT	°C	1055	Annual bio-temperature
PET	mm	1055	Potential evapotranspiration
MTWM	°C	1055	Mean temperature of the warmest month
MTCM	°C	1055	Mean temperature of the coldest month
WI	°C · month	1055	Warmth index
CI	°C · month	1055	Coldness index
**Humid/arid index**			
AP	mm	1055	Annual precipitation
PWQ	mm	1055	Precipitation of warmest quarter
PCQ	mm	1055	Precipitation of coldest quarter
**Integrative climatic index**			
AET	mm	1055	Annual actual evapotranspiration
NPP	g.a^−1^ · m^−2^	1055	Vegetation net primary productivity, calculated using the CASA model over each species’ range
Im	/	1055	Moisture index
**Traits**			
Seed mass (SM)	mg	1265	Weight of 1000 dry seeds
Growth form (GF)	/	1265	Classified into tree, shrub and liana.
Dispersal mode (DM)	/	1265	Classified into wind−, animal−, unassisted dispersal
Fruit type (FT)	/	1265	Classified into fleshy, dehiscent, indehiscent fruit
Breeding system (BS)	/	1265	Classified into hermaphrodite, monoecy, dioecy
Leaf habit (LH)	/	1265	Classified into deciduous and evergreen
Fruit development time (FDD)	day	880	Length of fruit ripen period, calculated as time between peak flowering phase and peak fruiting phase
Minimum juvenile period (MJP)	year	952	Time of first flowering and fruiting for a species

## Results

### Geographic pattern of seed mass

**Figure 1:**
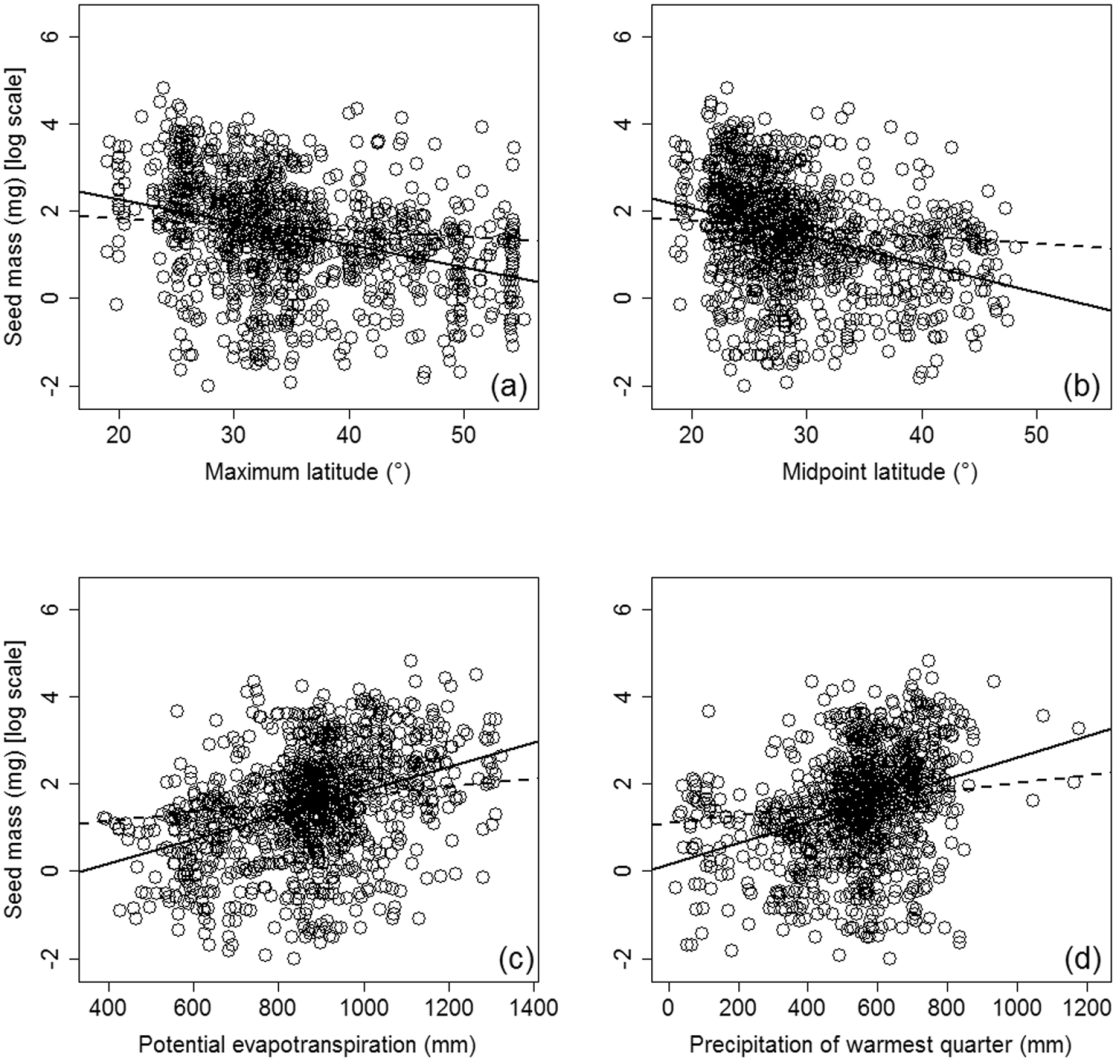
Associations between seed mass and maximum latitude, mid-point latitude, potential evapotranspiration and precipitation in warmest quarter, for 1055 woody species with coordinates and climate data available. Solid line represent result of linear regression, while dashed line represent that of phylogenetic model.

Seed mass decreased with latitude as inferred by the correlations of seed mass with the midpoint latitude and the maximum latitude of each species range, in both the phylogenetic and non-phylogenetic models (Table 2, Fig. 1a,b). However, the maximum latitude had slightly higher explanatory power than the midpoint latitude. On the other hand, a weak negative correlation was found between seed mass and the maximum longitude (R_phy_^2^ = 0.01, p = 0.003).

**Table 2:** Summary of univariate models for explaining seed mass with geographic, climate factors and other traits.

Predictor	Phylogenetic model				Non-phylogenetic model		
	p-value	R_phy_^2^	lambda	AIC	p-value	R^2^	AIC
**Geography gradient**							
Maximum longitude(MALO)	0.0247	**−** ***0***.***010***	0.963	1798.327	0.00278	−0.012	2397.640
Minimum longitude(MILO)	***0***.***1051***				<0.0001	−0.033	2381.966
Midpoint longitude(MPLO)	***0***.***6011***				***0***.***291***		
Maximum latitude(MALA)	<0.0001	−0.056	0.959	1784.701	<0.0001	−0.145	2292.898
Minimum latitude(MILA)	***0***.***1451***				< 0.0001	−0.082	2344.366
Midpoint latitude(MPLA)	0.0002	−0.052	0.959	1788.454	<0.0001	−0.136	2300.318
**Thermal index**							
MAT	<0.0001	0.059	0.958	1784.660	<0.0001	0.156	2283.788
MTCM	0.0001	0.055	0.959	1787.318	<0.0001	0.146	2292.526
MTWM	0.0334	***0***.***018***	0.962	1796.300	<0.0001	0.101	2329.516
ABT	<0.0001	0.068	0.958	1779.982	<0.0001	0.160	2280.400
PET	<0.0001	0.074	0.957	1787.341	<0.0001	0.153	2286.461
WI	<0.0001	0.073	0.957	1783.107	<0.0001	0.159	2281.404
CI	0.0324	***0***.***019***	0.961	1800.072	<0.0001	0.115	2317.894
**Humid/arid index**							
AP	0.0011	0.041	0.960	1799.999	<0.0001	0.144	2294.130
PCQ	***0***.***5577***				<0.0001	0.081	2345.762
PWQ	<0.0001	0.044	0.961	1788.819	<0.0001	0.120	2313.669
**Integrative climatic index**							
AET	<0.0001	0.071	0.958	1786.140	<0.0001	0.153	2285.953
Im	***0***.***3187***				<0.0001	0.078	2347.860
NPP	0.0258	***0***.***017***	0.962	1802.559	<0.0001	0.072	2352.168
**Traits**							
Growth form (GF)	<0.0001	0.053	0.960	1775.37	<0.0001	0.105	2328.519
Fruit type (FT)	<0.0001	0.079	0.958	1766.749	<0.0001	0.154	2287.839
Dispersal mode (DM)	<0.0001	0.110	0.965	1703.368	<0.0001	0.046	2374.651
Breeding system (BS)	0.0007	0.031	0.962	1784.995	<0.0001	0.035	2382.578
Leaf habit (LH)	0.0014	***0***.***014***	0.963	1786.937	<0.0001	0.101	2329.155
Fruit development time (FDD)	0.0246	0.028	0.960	1790.991	<0.0001	0.121	2313.541
Minimum juvenile period (MJP)	0.0001	0.033	0.961	1781.005	<0.0001	0.087	2340.605

Variables in bold italic fond were those with p > 0.05 or R_phy_^2^ < 0.02. In the R^2^ column, “−” denotes negative relationships while others were positive ones. This analysis is conducted with 725 species that have all the variables listed below.

### Associations between seed mass and climate factors

Out of the 13 climate indices, 11 were positively correlated with seed mass in the univariate phylogenetic models, whilst precipitation of the coldest quarter (PCQ) and moisture index (Im) were not (Table 2). Of the thermal indices, potential evapotranspiration (PET) had the highest correlation with seed mass (R_phy_^2^ = 0.074); whereas of the moisture indices, precipitation of the warmest quarter (PWQ) was the single best predictor (R_phy_^2^ = 0.044). Among the comprehensive climatic indices, however, actual evapotranspiration (AET) was better correlated with seed mass (R_phy_^2^ = 0.071) than NPP.

### Relationship between seed mass and other traits

Phylogenetic models showed significant effects of the seven life history traits on seed mass, with values of R_phy_^2^ varying from 0.01 to 0.11 (Table 2). Dispersal mode (DM), fruit type (FT) and growth form (GF) were the best single predictors for seed mass in phylogenetic models, while other traits had a R_phy_^2^ < 0.05.

**Figure 2:**
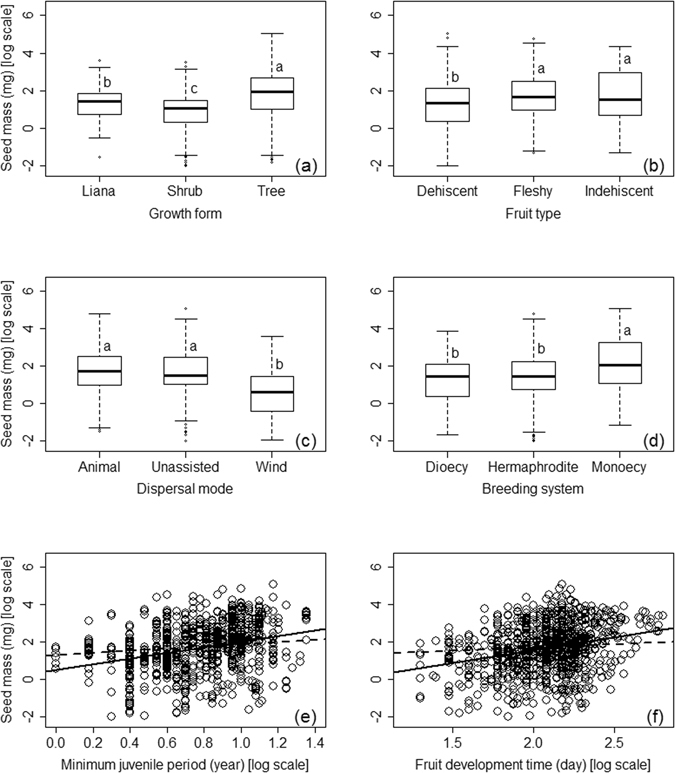
Associations between seed mass and (**a**) growth form, (**b**) fruit type, (**c**) dispersal mode, (**d**) breeding system, (**e**) fruit developing days and (**f**) minimum juvenile period in Chinese woody angiosperms. In (**a**) to (**d**), means with different letters were significantly different at *P* < 0.05. In (**e**,**f**), Solid line represent result of linear regression, while dashed line represent that of phylogenetic model. The analyses are conducted with all species available for each trait, with n = 1265 in (**a**) to (**d**), n = 935, 880 in (**e**,**f**) respectively.

As shown in Fig. 2, seeds of tree species were significantly heavier than seeds of lianas, which were heavier than those of shrubs. Species of animal dispersal and those without seed dispersal structures had significantly heavier seeds than species of wind-dispersal. Species that produce dehiscent dry fruit had lighter seeds than those with fleshy fruits and indehiscent dry fruit. Monoecious species had heavier seeds than species with dioecious and hermaphrodite breeding systems. Meanwhile, species with longer juvenile periods and fruit development time also tend to have larger seeds.

### Partitioning the effects of life history traits and environmental factors on seed mass

In the final phylogenetic model including only the environmental factors, only PET and PWQ were retained, which explained 8% of seed mass variance (Table 3); whereas in the final model including only the life history traits, five traits except LH and FDD were retained and the model’s R_phy_^2^ was 0.23. When environmental factors and life history traits were jointly used to explain seed mass, the three best phylogenetic models explained 27~28% of the variations (Table 4). Variance partitioning showed that the life history traits alone explained 22~23% of seed variance, while climate alone explained 7~8%, and the shared variation between environmental factors and life history traits was only 3% (Table 5). Taken together, the results suggested that climate explained much less variance in seed mass than life history traits.

**Table 3:** Summary of the best phylogenetic model for seed mass as a function of trait predictors alone, and climate as predictors alone. n = 725.

Trait only model			Environment only model		
Predictor summary	F-value	P-value	Predictor summary	F-value	P-value
Dispersal mode(DM)	54.869	<0.0001	Potential evapotranspiration (PET)	22.446	<0.0001
Fruit type (FT)	5.391	0.0046	Precipitation in warm quarter (PWQ)	5.941	0.0150
Growth form (GF)	13.750	< 0.0001			
Minimum juvenile period (MJP)	4.210	0.0399			
Breeding system (BS)	5.402	0.0046			
Model summary			Model summary		
lambda	0.955		lambda	0.963	
Total R_phy_^2^	0.234		Total R_phy_^2^	0.078	
AIC	1661.516		AIC	1767.849

**Table 4:** Summary of the best multivariate phylogenetic models for seed mass as a function of traits and climate together. n = 725.

**Predictor summary**	Top-1 model		Top-2 model		Top-3 model	
	F-value	P-value	F-value	P-value	F-value	P-value
Dispersal mode(DM)	55.886	<0.0001	49.923	<0.0001	55.642	<0.0001
Fruit type (FT)	3.764	0.0236	4.141	0.0163	3.709	0.0250
Growth form (GF)	15.179	<0.0001	11.025	<0.0001	15.009	<0.0001
Minimum juvenile period (MJP)	4.085	0.0437	—	—	—	—
Breeding system (BS)	7.106	0.0009	5.971	0.0027	7.038	0.0009
Potential evapotranspiration (PET)	11.902	0.0006	28.630	<0.0001	12.385	0.0005
Precipitation of warmest quarter (PWQ)	—	—	6.888	0.0089	—	—
**Model summary**						
lambda	0.948		0.950		0.950	
AIC	1651.865		1652.966		1653.276	
Total R_phy_^2^	0.280		0.270		0.269

**Table 5:** Variance partitioning for the effect of traits and climate in explaining seed mass. n = 725.

	Model	Climate R_phy_^2^	Trait R_phy_^2^	Shared R_phy_^2^	Unexplained R_phy_^2^
Top-1 model	SM~DM + FT + GF + MJP + BS + PET	0.074	0.234	0.028	0.720
Top-2 model	SM~DM + FT + GF + BS + PET + PWQ	0.078	0.221	0.029	0.730
Top-3 model	SM~DM + FT + GF + BS + PET	0.074	0.221	0.026	0.731

Note: SM: Seed mass; DM: Dispersal mode; GF: Growth form; MJP: Minimum juvenile period; BS: Breeding system; PET: Potential evapotranspiration; PWQ: Precipitation of warmest quarter.

## Discussion

### Latitudinal patterns of seed mass and the effect of climate

Since the Salisbury’s seminal review (1942), factors influencing the diversity of seed sizes among species and regions have remained an intriguing but only partially answered question^23, 24^. At a broad scale, the most striking pattern is the latitudinal decrease of seed mass^3, 5, 9^. However, many previous studies at large scales did not examine the effects of phylogeny on geographic seed mass patterns. It remains unclear whether the latitudinal seed mass pattern still exists after the phylogenetic relatedness among species has been accounted for. This is important because phylogenetic relatedness will lead to non-independence of data^25^, and thus, we cannot reject the possibility that a significant correlation between seed mass and latitude (and climate, other traits) may be simply caused by data non-independence (which is well known that may inflate significance and R^2^, for an example see Sun *et al*. 2017). In this study, through explaining seed mass with both linear model and PGLS, we showed that seed mass of Chinese woody plants did decrease significantly with latitude, even in models where the effects of phylogeny have already been accounted for. Thus our results provide sound supports to previous regional and global studies for the latitudinal decrease of seed mass^5, 9, 11, 26^. We also showed that the R^2^s of phylogenic models were mostly much lower than that of non-phylogenic models (Table 2); this is not only true for the geographic variables but also for climate indices and most life history traits. This is consistent with our prediction that phylogeny plays an important role in shaping the observed latitudinal gradients in seed mass (and the correlations of climate and other traits with seed mass). In another word, the latitudinal decrease of seed mass is not simply a result of climate gradient and covariation with other life history traits. Instead, evolutionary history also plays a key role.

In the global study of Molles *et al*. (2007), seed mass deceased with latitude at a rate of 1.10% per degree. In this study, the linear relationship between seed mass (y) and mid-point latitude (x) was in the form of log_10_(y) = 3.38 − 0.065x. This means that the latitudinal decrease rate of seed mass was 1.16% per degree in China, surprisingly similar to that estimated from the global dataset. Whether this suggests that there is a universal pattern of seed mass, irrespective of differences in flora among continents, deserves further examination. In Molles *et al*. (2007), the R^2^ between seed mass and latitude was 0.24, clearly higher than the linear model R^2^s in our analysis (0.08~0.15, Table 2). This is not surprising because they used data for 11,481 species × site combinations, with exactly known latitudes (and other variables) of the sites. Here we used the maximum, minimum and mid-point latitude (and mean climate indices) across species’ ranges, which may be a major reason why the R^2^s in this study were not only low for latitude but also for climate variables. However, this provides us an opportunity to compare the R^2^s of maximum, minimum and mid-point latitude in explaining seed mass. Our results showed that the explanatory power was highest from the maximum latitude, followed by the mid-point and the minimum latitude. This suggests that the temperature at the northern limit of species’ range is more powerful in affecting geographic seed mass patterns than that at the southern limit. Stahla *et al*. (2014) also found strong effect of range limit on seed mass for 250 tree species in North America, which they suggest is that because some woody plants with large seed mass did not occur in cold climates^13^.

The marked change in seed mass along latitudinal gradient suggests the influence of climate on seed mass^5^. Our results showed that seed mass was positively correlated with most climate variables in phylogenetic models (Table 2), confirming previous studies that seed mass is generally higher under favorable climates^9, 13^. We found that PET was the best single predictor out of the thermal indices, while precipitation in the summer (PWQ) was the best predictor in the moisture indices, as indicated by the R_phy_^2^ in Table 2. Further, PET and PWQ were the only variables retained in the final phylogenetic models (Tables 3 and 4). These results are consistent with the fact that energy and water availability in the growing season (instead of mean temperature and precipitation of the year, or that in the winter) are mainly responsible for biological activity, and thus are closely related to many large-scale patterns such as productivity and biodiversity^27^. Molles *et al*. (2007) found that NPP was included in the final model explaining global seed mass pattern. However, here we show that NPP is a weaker predictor (R_phy_^2^ = 0.017) compared with PET and PWQ. It is possible that climate not only affects seed mass through NPP, but also exerts other influences. For instance, seed mass are hypothesized to be influenced by growing season length and abundance of vertebrate seed dispersal agents^28^, both are higher at lower latitudes with high PET and PWQ. As for the relative importance of energy vs. water availability, our results showed PET was far more powerful than PWQ in multivariate models (as indicated by the F values in Tables 3 and 4). This suggests that energy is more important for the geographic seed mass patterns, at least for the woody angiosperms in China.

### The role of life history traits

Climate only explained a small proportion of variation in seed mass (Table 3), which is also observed in other studies^12^. Consequently, we went further to test whether the large variations in seed mass across species are caused by the covariation of seed mass with other life history traits^2, 23, 28^. We found that dispersal mode and growth form had much higher importance (F value) than other traits in multivariate models (Tables 3 and 4), supporting previous studies that the two traits were key for seed mass. The effects of these two traits are also evident in Fig. 2, which showed that seed mass was significantly larger for trees than the liana and shrub growth forms, and animal dispersed seeds were clearly heavier than wind-dispersed ones^19^.

As stated in the introduction, our dataset included some reproductive traits that has seldom been tested in large-scale seed mass studies. Considering that seed mass was found to be related to these traits in the local scale studies^2, 5, 29–33^, we had expected that the R^2^ of multivariate models would be markedly improved by these additional traits. However, we found that while most of these traits did show significant correlations with seed mass (Table 2), they did not contribute much in both the “trait only” phylogenetic model (Table 3) and the “trait-and-climate” models (Table 4). Though our expectation was not proven to be the case, this may turn out to be a positive news. We showed that the key traits affecting geographic seed mass patterns in China (growth form and dispersal mode) were similar to those found in global studies (e.g. Moles *et al*. 2007), even when many additional traits were considered simultaneously. This may suggest that the biotic mechanisms underlying broad-scale seed mass patterns are universally consistent.

However, we still suggest future studies to test the effects of reproductive traits on seed mass, before drawing a conclusion. For instance, fruit type and dispersal mode are different traits (note that fruit type is categorized based on fruit morphology, while dispersal mode is classified by dispersal agents^34, 35^, but their roles were not well distinguished in some studies^30^. Fruit development time^5^ and minimum juvenile period^20^ were also suggested being potentially important factors influencing seed mass. It is possible that these traits are important in some regions and taxa.

### The relative effect of climate and life history traits on seed mass

Through variance partitioning (Table 5), we showed that seed mass was by far more explained by life history traits than climate, which is consistent with previous studies. In a comparative study on five distinct temperate floras from three continents^26^, seed mass ranged at least five orders of magnitude within each flora. However, the difference between floras accounted for only 4% of the variation in seed mass between species, suggesting a rather weak role of environmental gradients on seed mass. Using a global database, Moles *et al*. (2005) also found that climate variables had much less explanatory power than seed dispersal mode and growth form^28^. Here we further showed that the shared variations explained by climate and life history traits were only 3%. This suggests that climate and life history traits influenced seed mass largely in an independent way. Similar to previous large-scale studies, there are large proportions of variations in seed mass that were not explained in this study (Table 5), even when many climate indices and life history traits were included. One possibility is that the ecological interactions among species within-site are mainly responsible for the unexplained variations^9^. Meanwhile, there are still many other mechanisms that may affect seed mass, including soil and light, etc.^4, 5^. Further studies are needed to test these mechanisms with climate and life history traits together, for a better understanding of large-scale seed mass patterns.

## Methods

### Data collection

Data on seed mass (dry seed mass per 1000 seeds, mg) for angiosperm woody plant species were compiled mainly from two books: Seed of Woody Plants in China^36^ and Seed and fruits of Woody Plant in China^37^. In the two books, maximum and minimum seed mass values were recorded for 1222 and 627 woody species across the country respectively, and we used the mid-value of the seed mass range to represent the average seed mass for each species. In addition, seed mass data were also collected from papers published up to December 2015^38–50^. For data from these sources, when the authors provided a single seed mass value for a species, the value was adopted. When multiples values were reported for a species from different sources, we used the mean value. When only the seed mass ranges were reported, we used mid-value. All the species names were verified with the Plant List (http://www.theplantlist.org/) to correct synonyms, while variants were removed. In total we obtained seed mass data for 1265 woody angiosperms species.

Seven life-history traits in addition to seed mass for these species were also collected whenever available from various sources (see below); these traits included growth form (GF), leaf habit (LH), fruit type (FT), propagule dispersal mode (DM), breeding system type (BS), the period between peak flowering and peek fruiting (fruit development time) (FDD), and the time to first year of flowering and fruiting (minimum juvenile period) (MJP) (Table 1). The first five traits were recorded as categorical variables, obtained mainly from Flora of China (http://www.eflora.cn/), Chinese Trees^51^ and Chinese Higher Plant^52^. Growth form includes three categories (tree, shrub and liana), whereas leaf habit was divided into deciduous and evergreen with the latter including a small fraction of semi-evergreen species. Dispersal mode of propagules was grouped into three types including wind-, animal- and unassisted dispersal^42, 53^. We did not use the five to eight dispersal categories adopted by some researches^2, 34^, because there was not enough information in most Chinese sources. Approximately half of the dispersal mode data were extracted from Chinese literature (Appendix 1) and the seed information database of Kew (http://data.kew.org/sid/), while for the rest, species were assigned to a dispersal mode base on fruit and seed ornamentation and appendages^53^ using the information available in the literature^36, 37, 51, 52^. For species with multiple dispersal methods, only the most common mode was used. For fruit types, three categories were used, i.e., fleshy, indehiscent (dry fruit remaining closed when ripe), dehiscent (dry fruit opening when ripe)^35, 54^. Breeding system includes three categories^55^, i.e., hermaphrodite, dioecy (including gynodioecy, androdioecy and triodioecy), and monoecy (including gynomonoecy and andromonoecy). We were able to obtain species-level breeding system information for most species (~80%), and genus-level information was used for the remaining species. In addition to these five categorical traits, two quantitative traits, i.e., fruit development days, and minimum juvenile period, were extracted from the two books^36, 37^ when available. Among the seven trait groups, for the five categorical traits we had data for each of the 1265 species, while for the other two quantitative traits we had data for most but not all species (70–75%) (Table 1).

As the sampling locations were not recorded for most of the species in the database, climate data over the natural distribution range in China for each species were retrieved as environment variables of the species. Among the 1265 species in our database, climate information for 1055 species over their range were extracted from the Atlas of Woody Plant in China^56^. The 1055 species’ range were 18.2 °N~55.3 °N and 73.5 °E ~135 °E. The book has provided mean values for thirteen climatic variables across the range of each species (Table 1), as well as the species’ distribution map at the county level. The climate variables include three groups as follows: (1) The thermal indices: mean annual temperature (MAT, °C), annual bio-temperature (ABT, °C)^57^, potential evapotranspiration (PET, mm), the warmth index (WI, °C · month) and coldness index (CI, °C · month) of Kira^58^, mean temperature of the warmest month (MTWM, °C), and mean temperature of the coldest month (MTCM, °C). (2) The humid/arid indices: mean annual precipitation (AP, mm), precipitation in the warmest quarter of the year (PWQ, mm) and precipitation in the coldest quarter of the year (PCQ, mm). (3) The integrative indices include annual actual evapotranspiration (AET, mm) and moisture index (Im)^59^, and vegetation net primary production (NPP, g.a^−1^.M^−2^) estimated using the CASA model (for details, see Fang *et al*. 2009). In addition, maximum and minimum latitude and longitude for each species were also extracted from the distribution map to calculate the latitude and longitude midpoints of each species’ range. Longitudinal and latitudinal range were also calculated to explore their correlation with seed mass, however, we did not include this into this paper.

### Data analyses

Seed mass, fruit development days, and minimum juvenile period were log_10_-transformed (the other four traits were categorical variables), to increase normality in the data, before statistical analyses were performed with R.3.2^60^. One-sided Wilcoxon rank sum test was used to compare median between two groups in the four categorical traits by wilcox.test function in R. We conducted phylogenetic analyses to account for the possible influence of phylogeny on the results in explaining the relationship of seed mass with other traits and environmental factors. For phylogenetic analysis, we first built the phylogenetic tree for 1265 species in this study using the most updated phylogeny of plants^61, 62^. The phylogeny of Zanne *et al*. (2014) was generated using sequence data of seven gene regions available in GenBank, as well as fossil data^61^. Qian & Jin (2016) updated this phylogeny and provided an R-code (the ‘S.PhyloMaker’ function) to generate specific phylo-trees by user-defined species list, which then calculates the branch lengths of the phylo-trees using BLADJ algorithm^62^. Based on the phylo-tree we built (Appendix 2), phylogenetic signal of quantitative traits were calculated as Blomberg’s K-value using the ‘phylosignal’ function in the R package ‘phytools’^63^, and Phylogenetic generalized least square method (PGLS) was used to build regression models in phylogenetic context^64^ with the ‘gls’ function in R package ‘nlme’^65^.

We first conducted bivariate analyses to examine the relationship of seed mass with each of the life history traits and environment variables. We then used multivariate analyses to explain seed mass variation with regard to other life history traits alone, environment alone, and traits and environment together using a subset of the data consisting of 725 species with values for all seven traits and 13 environment variables (6 thermal indices, 4 humid/arid indices, and 3 integrative climate indices). There were 20 variables as potential predictors of seed mass, thus we needed to reduce the number of predictors to minimize collinearity. We did a three-step procedure to estimate the combined effect of climate and life history traits on seed mass. Taking the climatic variables group as an example, as most of the climatic variables are closely inter-correlated (Appendix 3), we started by reducing the initial pool of 13 climatic variables to a smaller number based on the results of the bivariate analysis^66^. First, the variables with an R^2^ < 0.02 in explaining seed mass were excluded (see Table 3). Next, within each of three groups of climate indices, we used AIC-based backward elimination to select the variables that best explained seed mass variation. We also applied this procedure to all life history traits as a group. Lastly, all traits and environment predictors that survived the first two steps were included in building the full models (Table 4), and AIC was used to select the most parsimonious model. As there are too many possible interactions terms that would overwhelm our available degrees of freedom in the full models, we did not include any interactions. Determination coefficients (R_phy_^2^) for each model were calculated using the maximum likelihood (ML) estimation^67^. In the context of phylogeny analysis, R_phy_^2^ is defined as the proportion of variation explained by the linear model, taking the correlation among observations into account, divided by that of the null model (intercept-only) with the same correlation structure in PGLS^68^, thus R_phy_^2^ may be regarded as a pseudo-R^2^. To partition effects of life history traits vs. climatic factors on seed mass, the overlaid contribution was calculated as sum of the R_phy_^2^ from the environment group and trait group minus the R_phy_^2^ from full model.

Here, in multivariate analyses we present results of phylogenetic models using a subset of the data consisting of 725 species for which all traits and environmental factors were available. Meanwhile, the full dataset was used in the bivariate analyses. We also provide results from non-phylogenetic models for better comparison to previous studies.

## Electronic supplementary material

Appendice 1-3
