# Supplementary material for: Seed mass of angiosperm woody plants better explained by life history traits than climate across China
Source: Sci Rep. 2017 Jun 2;7:2741. doi: 10.1038/s41598-017-03076-2 (PMC5457422; doi:10.1038/s41598-017-03076-2)

APPENDICES

Seed mass of angiosperm woody plants were better explained by life history traits than climate across China

Authors: Jingming Zheng*, Zhiwen Guo, Xiangping Wang

Appendix 1 Literatures for dispersal syndrome assignment of angiosperm woody plants in China

1 Xiao, L. & Pu, Z. Study on the relationship between the spread of Loranthaceae and birds in Xishuangbanna, Yunnan. *Acta Ecologica Sinica* **14**, 128-135 (1994).

2 Wu, D. & Wu, Y. Regeneration of *Phoebe bournei* Yang population. *Journal of plant resources and environment* **7**, 8-12 (1998).

3 Ran, J., Xiong, Z. & Zhang, C. Preliminary study on the fruits eating birds on seed dispersal in Maolan Karst forest area. *Journal of Guizhou Normal University (Natural Science)* **17**, 63-66 (1999).

4 Wang, W. & Ma, K. Predation and dispersal of *Quercus liaotungensis* acorns by Chinese rock squirrel and Eurasian jay. *Acta Botanica Sinica* **41**, 1142-1144 (1999).

5 Wang, W., Ma, K. & Gao, X. Spatial and temporal patterns of *Quercus liaotungensis* acorn predation by vertebrates in Dongling Mountain, northern China. *Acta Botanica Sinica* **42**, 289-293 (2000).

6 Wang, Z. *et al. Morus Macroura* and the wildlife who eat their beery in different mountain regions, Xishuangbanna, Yunnan. *Journal of Mountain Science* **18**, 267-271 (2000).

7 Wang, Z. *et al.* The relationship between *Choeorpsondias axillarsi* and wildlife in Xishuangbanna. *Journal of Northeast Forestry University* **28**, 55-57 (2000).

8 Li, X., Yin, X. & He, S. Seed dispersal by frugivorous birds in Nanjing Botanical Garden Mem. Sun Yat-Sen in autumn and winter. *Biodiversity science* **9**, 68-72 (2001).

9 Li, X., Yin, X. & He, S. Tree fruits eaten by birds in Nanjing Botanical Garden Mem. Sun Yat-Sen in autumn and winter. *Chinese Journal of Zoology* **36**, 20-24 (2001).

10 National Seed and Seedling Center. Seeds of woody plants in China. *Beijing: Chinese Forestry Publishing House* (2001).

11 Shangguan, T. & Zhang, F. The endangered causes of *Elaeagnus mollis*, an endemic to China. *Acta Ecologica Sinica* **21**, 502-505 (2001).

12 Wang, G. & Zhou, G. Correlation analysis on the relationship between plant life form, fruit type and hydrothermic factors in Gansu woody plant flora. *Bulletin of botanical research* **21**, 448-455 (2001).

13 Wang, Z. *et al.* Bird diversity during ripe berry period of *Morus mocrora* in mountain region, Xishuangbanna, Yunnan. *Journal of Mountain Science* **19**, 48-52 (2001).

14 Wang, Z. *et al.* Study on Bird Diversity and Frugivorous Birds in Fallow Succession Forest Regions of Mengsong, Xishuangbanna. *Zoological Research* **22**, 205-210 (2001).

15 Wu, D. & Wang, B. Seed and seedling ecology of the endangered *Phoebe bournei* (Lauraceae). *Acta Ecologica Sinica* **21**, 1751-1760 (2001).

16 Zhang, Z., Cao, M., Yang, X. & Zhao, Z. *Codariocalyx motorius* seed removed by ants in Xishuangbanna. *Acta Ecologica Sinica* **21**, 1847-1853 (2001).

17 Zhang, Z. & Wang, F. Effect of rodents on seed dispersal and survival of wild apricot (*Prunus armeniaca*). *Acta Ecologica Sinica* **21**, 839-845 (2001).

18 Liu, Y., Chen, J., Bai, Z., Deng, X. & Zhang, L. Seed dispersal, seed predation, and seedling spatial pattern of *Garcinia cowa* (Guttiferae). *Acta Phytoecologica Sinica* **26**, 427-434 (2002).

19 Wang, Z. *et al. Trema orientalis* seeds dispersed by birds and its ecological role. *Zoological research* **23**, 214-219 (2002).

20 Lu, C. Biology of mistletoe (*Viscum coloratum*) and its seed dispersal by frugivorous birds. *Acta Ecologica Sinica* **23**, 834-839 (2003).

21 Xiao, Z., Zhang, Z. & Wang, Y. Rodent's ability to discriminate weevil-Infested acorns: potential effects on regeneration of nut-bearing plants. *Acta Theriologica Sinica* **23**, 312-320 (2003).

22 Xiao, Z., Zhang, Z. & Wang, Y. Observations on tree seed selection and caching by Edward's long-tailed rat (*Leopoldamys edwardsi*). *Acta Theriologica Sinica* **23**, 208-213 (2003).

23 Li, X. & Yin, X. Seed dispersal by birds in Nanjing Botanical Garden Mem. Sun Yat-Sen in spring and summer. *Acta Ecologica Sinica* **24**, 1452-1458 (2004).

24 Lu, C., Chang, J. & Xu, Q. Regeneration of *Phellodendron amurense* and its deed dispersal by frugivorous birds. *Chinese Journal of Ecology* **23**, 24-29 (2004).

25 Ma, J., Li, Q., Sun, R. & Liu, D. Rodents as the key predators of ground seeds of *Quercus liaotungensis* in Xiaolongmen Forestry Centre, Beijing. *Zoological Research* **25**, 287-291 (2004).

26 Wei, M., Chen, Z., Ren, H., Zou, F. & Yin, Z. Seed dispersal of the pioneer shrub *Rhodomyrtus tomentosa* by frugivorous birds and ants. *Biodiversity Science* **12**, 494-500 (2004).

27 Xiao, Z. & Zhang, Z. Small mammals consuming tree seeds in Dujiangyan forest. *Acta Theriologica Sinica* **24**, 121-124 (2004).

28 Xiao, Z., Zhang, Z., Wang, Y. & Cheng, J. Acorn predation and removal of *Quercus serrata* in a shrubland in Dujiangyan Region, China. *Acta Zoologica Sinica* **50**, 535-540 (2004).

29 Gao, R., Liu, T., Zhang, H., Han, Y. & Cong, L. Study on relationship between *Planta lnose* fruit types and environment succession in DaQing Clough. *Journal of arid land resources and environment* **19**, 174-178 (2005).

30 Lu, X. *et al.* Fruits foraging patterns and seed dispersal effect of frugivorous birds on *Hippophae rhamnoides* subsp. *sinensis*. *Chinese Journal of Ecology* **24**, 635-638 (2005).

31 Ma, J. Effects of *Cyanopica cyana* ingestion on seed germination of *Hippophae rhamnoides* subsp. *sinensis*. *Acta Bot. Boreal-Occident Sin.* **25**, 472-477 (2005).

32 Cao, L., Xiao, Z., Zhang, Z. & Guo, C. Patterns of seed predation and removal of Cerasus pseudocerasus by rodents in a subtropical forest, Sichuan. *Chinese Journal of Zoology* **41**, 27-32 (2006).

33 Du, Y., Peng, S., Huang, Z. & Xu, G. Analysis of seed death of *Castanopsis chinensis* in the dispersal process in Dinghushan biosphere reserve. *Ecology and environment* **15**, 1284-1288 (2006).

34 Li, X., Yin, X., Xia, B., Li, W. & Li, Y. Effects of bird seed dispersal on diversity of the invaded plants in several hedge types. *Acta Ecologica Sinica* **26**, 1657-1666 (2006).

35 Zhang, T. *et al.* Seed predation and dispersal of *Castanopsis fargesii* by rodents in Tiantong

Mountain, Zhejiang Province. *Chinese Journal of Ecology* **25**, 161-165 (2006).
36 Zhang, Z. Effectiveness of ants in dispersal of seed of *Codariocalyx motorius* and preventing the seed from rodents' predation. *Scientia Silvae Sinicae* **42**, 58-62 (2006).
37 Chen, X., Tian, F. & Qi, P. Composition and Vertical Differentiation of Fruit Types in Baishuijiang National Nature Reserve in Gansu Province. *Scientia Silvae Sinicae* **43**, 61-66 (2007).
38 Tang, Z., Sheng, L., Ma, X., Cao, M. & Zhang, S. Fruit consumption and seed dispersal of *Morus macroura* by two frugivorous bats in Xishuangbana, southeast China. *Acta Ecologica Sinica* **27**, 1895-1902 (2007).
39 Tian, Z., Sheng, L., Ma, X., Cao, M. & Zhang, S. The effect of ingestion by bat (*Rousettus leschenaulti*) on seed germination of *Ficus racemose* and *Ficus hispida* (Moraceae). *Acta Ecologica Sinica* **27**, 1343-1349 (2007).
40 Wan, W., Zhang, H. & Zhang, Z. Effects of predation risk on cultivated walnut (*Juglans regia*) seeds hoarding behavior by David's rock squirrel (*Sciurotamias davidianus*) in enclosure. *Acta Theriologica Sinica* **27**, 358-364 (2007).
41 Wang, B. & Yang, X. Seed Predation of *Apodemus latronumon* on 18 Plant Species in Northwest Yunnan. *Zoological Research* **28**, 389-394 (2007).
42 Fan, P., Huang, B. & Jiang, X. Seed dispersal by black crested gibbons *(Nomascus concolor*) in the Wuliang Mountains, Central Yunnan. *Acta Theriologica Sinica* **28**, 232-236 (2008).
43 Liu, X. *et al.* Feeding and hoarding strategies of Tanezumi rat (*Rattus tanezumi*). *Journal of Qufu Normal University* **34**, 97-100 (2008).
44 Li, X. Ecological significance of bird perches on the restoration of forest vegetation. *Acta Ecologica Sinica* **29**, 4448-4454 (2009).
45 Li, X., Dong, Y., Xu, S. & Huang, B. Role of birds in seed dispersal of *Phoebe hunanensis* in Sppirit Vally forest of Nanjing. *Chinese journal of ecology* **28**, 32-37 (2009).
46 Wang, J., Du, G., Cui, X., Zheng, X. & Qi, W. Germination characteristics of 61 woody species from the eastern Qinghai-tibet Plateau of China and their life history correlates. *Chinese Journal of Plant Ecology* **33**, 171-179 (2009).
47 Chen, L., Wu, Y., Chen, D. & Xing, F. Study on the seed dispersal and secondary succession of Shek Kwu Chau, Hong Kong. *Guihaia* **30**, 651-656 (2010).
48 Jiang, M., Cao, L., Xiao, Z. & Guo, C. Frugivorous birds and its impacts on seed dispersal of wild cherry (*Prunus pseudocerasus*) in a Dujiangyan forest, China. *Chinese journal of zoology* **45**, 27-34 (2010).
49 Yang, X., Tang, Y. & Cao, M. Diaspore traits of 145 species from a tropical seasonal rainforest in Xishuangbanna, SW China. *Acta Botanica Yunnanica* **32**, 367-377 (2010).
50 Zhang, X., Xu, J., Shen, H. & Huang, J. Animal predation and dispersal of *Sorbus pohuashanens* fruits and seeds. *Chinese Journal of Applied Ecology* **21**, 2677-2683 (2010).
51 Chang, G. & Tai, F. The effects of seed size and germination schedule on the hoarding strategy of David's rock squirrel *Sciurotamias dabidianus*. *Journal of Northwest University (Nature Science)* **41**, 843-847 (2011).
52 Chen, Y., Wang, J., Zhang, H., Ding, H. & Tang, S. Observation on feeding habit of dominant birds and forage sites in an evergreen broadleave forest, Tiantong Forest Park, Zhejiang. *Chinese journal of Zoology* **47**, 46-53 (2012).
53 Lu, S. *et al.* Traits of fruit, seed and seedling growth of karst native tree species *Itoa orientalis*. *Guihaia* **32**, 637-643 (2012).

54 Shi, Y., Li, X. & Guo, Z. Tree fruits consumed by *Eophona migratoria* in winter. *Journal of biology* **29**, 20-23 (2012).

55 Yu, S., Fang, W. & Zhang, X. Fruit type and their altitudinal distribution patterns of wild plants in Beijing. *China Journal of Ecology* **31**, 2529-2533 (2012).

56 Li, J., Guo, C. & Xiao, Z. Fruit composition and seed dispersal strategies of woody plants in a Dujiangyan subtropical forest, Southwest China. *Biodiversity science* **21**, 572-581 (2013).

57 Ruan, H., Bai, B., Li, N., Pan, Y. & Lu, C. Fruit diet, selectivity and seed dispersal of Hatinh langur (*Trachypithecus francoisi hatinhensis*). *Acta Ecologica Sinica* **33**, 110-119 (2013).

58 Yu, F., Shi, X., Yi, X. & Wang, D. Effects of relative abundance of *Quercus mongolica* acorns on five tree species seed dispersal in Xiaoxing'an Mountain, Northeast China. *Chinese Journal of Applied Ecology* **24**, 1531-1535 (2013).

59 Zhou, L., Yang, X., Wang, J. & Zhou, Y. Predation and removal of rodents on the seeds with different size and pericarip traits. *Chinese Journal of Applied Ecology* **24**, 2325-2332 (2013).

60 Yang, X. & Gong, H. Seed predation by rodents in northwest Yunnan Province. *Journal of Zhejiang Agriculture and Forestry University* **32**, 440-445 (2015).

Appendix 2 Phylotree for 1265 woody Angiosperm

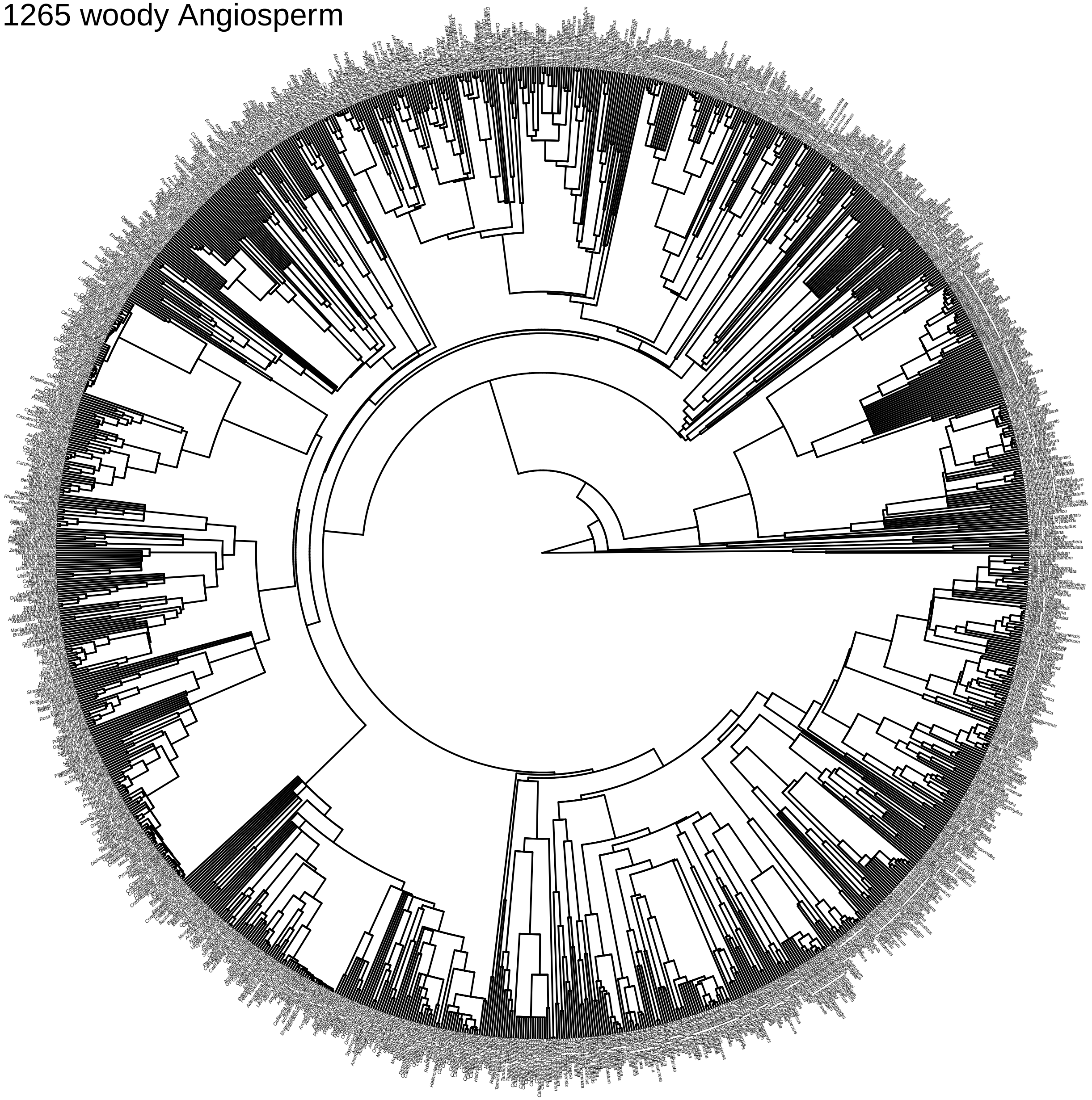

Appendix 3 Pearson correlation coefficients among climatic variables in China. n=1055

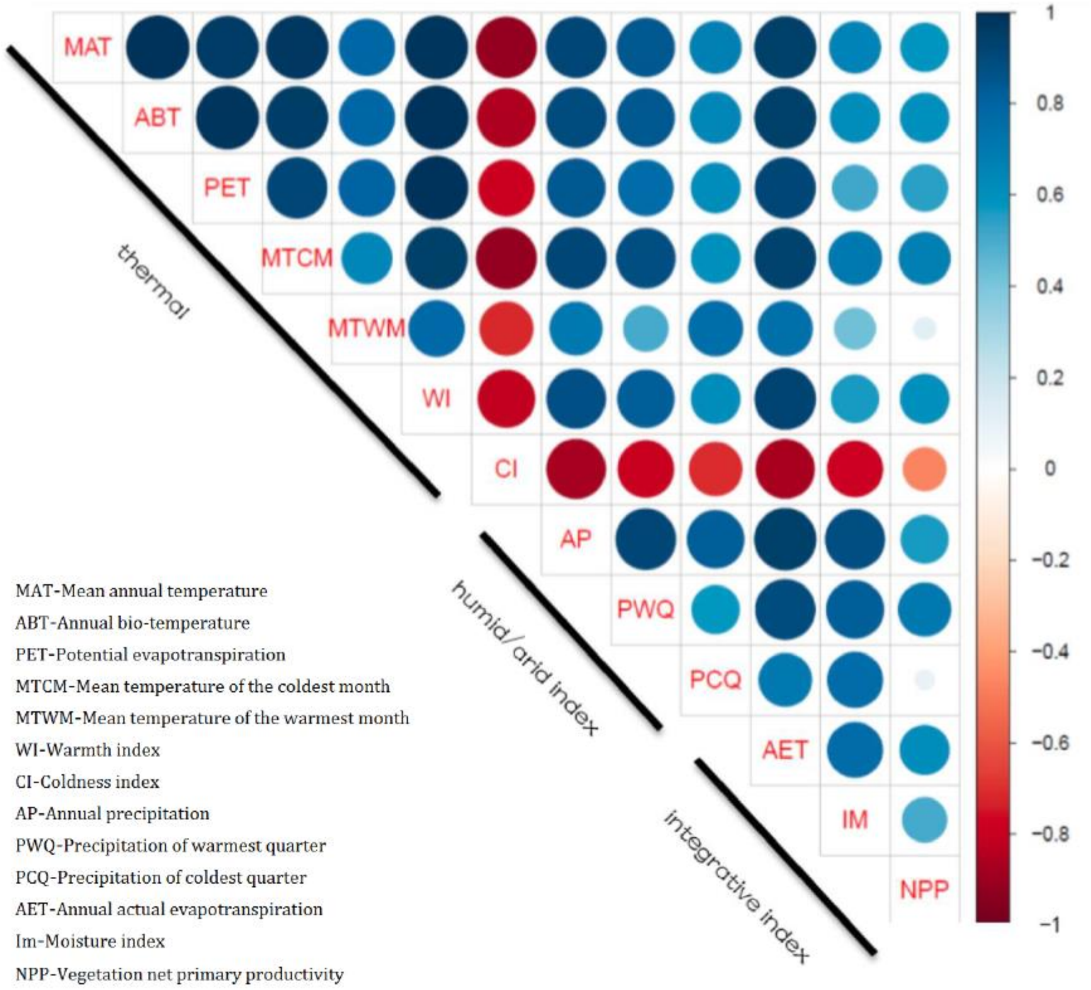

Supplement: Supplementary file 1 — Appendice 1-3 [file 41598_2017_3076_MOESM1_ESM.pdf]
